# Research on production capacity planning method of open-pit coal mine

**DOI:** 10.1038/s41598-023-35967-y

**Published:** 2023-05-29

**Authors:** Guangwei Liu, Weiqiang Guo, Senlin Chai, Jiaming Li

**Affiliations:** 1 School of Mining, Liaoning Technical University, Fuxin, 123000 China; 2 School of Economics & Management, Yancheng Institute of Technology, Yancheng, 224051 China

**Keywords:** Energy science and technology, Engineering

## Abstract

Reasonable production capacity is related to the economic benefits of an open-pit coal mine. This study analyzes the relationship between the working face length, the annual advancing speed and the production capacity. It constructs a production capacity function relationship model. Take the Baorixile open-pit coal mine as an example. The remaining unmined parts are divided into four regions, and the range of production capacity in each region is analyzed by the established model and the determined respectively. On this basis, three mining district division plans are proposed. By analyzing and comparing the stripping ratio, mining life of the district, fault influence, difficulty of transition connection in the mining districts, the convenience of transportation system layout and other indexes of each plan, Plan 3 is determined to be the optimal plan. The production capacity planning results of each mining district in this plan are as follows: the production capacity of the 3rd mining district is 30–35 Mt/a; the production capacity of the 4th mining district in Region 1 is 20–31 Mt/a, and the production capacity in Region 2 is 24–33 Mt/a; the production capacity of the 5th mining district is 20–27 Mt/a.

## Introduction

China is heavily dependent on coal, and the proportion of coal in the energy structure will be continuously high^1–3^. The open-pit coal mining has the characteristics of high recovery and high safety factors, open-pit coal mining can bring huge economic benefits to the mining area^4–7^. In the mining design of open pit coal mine, planning reasonable production capacity is an extremely important factor to be decided. Many production factors are determined around production capacity. Therefore, determining a reasonable production capacity can reduce the production costs of mining enterprises, so that the social and economic efficiency of mining enterprises increased, thereby enhancing competitiveness of enterprises. However, due to the large range of open-pit mines, it is necessary to divide mining districts for mining, and the division of mining districts affects the planning of production capacity^8,9^. At the same time, may also be due to an unreasonable mining district division plan caused by different working face lengths, so that the production capacity cannot meet the approved requirements, seriously affecting the economic benefits of the mine. Production capacity is the core of many decision-making factors in mines, which affects the scale of mine investment, cost and economic benefits^10–12^. Others such as the general layout, the number of the labour force, and the construction of external transportation corridors are planned based on the production capacity. It is generally tough to modify once a decision has been made. As a result, it is critical to accurately determine production capacity^13,14^. The research of open-pit coal mining district division and production capacity planning for mining businesses' economic advantages is of great significance^15–18^.

Li^19^ developed a theoretical approach to the optimization of mine life and design capacity. The model is developed based on marginal analysis. The model solves for the production rate at which the present value of marginal costs equals the present value of marginal revenues-the rate that microeconomic theory shows will maximize the net present value of production from the mine. The proposed model maximizes the net present value of cash flows over the life of an operation. Elkington et al.^20^ outlined a new MIP method of simultaneously optimizing mining and processing capacities. The case study demonstrated how the cut-off grade and stockpiling practices of the operation affects the optimal production capacities, as well as NPV. Zhang et al.^21^ considered resource reserves, production technology, and economy as constraints on the production capacity of open-pit mines. To maximize profit, they constructed a reasonable revenue function for a single pit and established a multi-scale pit production model using an integer programming method. The majority of optimization models for finding production capacities ignores expected variations and uncertainty in metal content or the available supply of ore and waste material. An extension to an established theory of cut-off grade is proposed by Asad and Dimitrakopoulos^22^ to determine the optimal production capacities based on a stochastic framework relying on multiple grade-tonnage curves derived from a set of simulated orebody realizations. To realize economic dynamic assessment and optimization of production capacity of an open-pit coal mine with the balance of the total amount of stripping, Zhang et al.^23^ combined optimization of the final pit with production capacity and adopted the Milawa algorithm to quickly generate multiple final pit plans. The final pit plan, mine production capacity and stripping sequence are determined with optimally dynamic economic indicators, and this research method was applied in Dongjielegele Iron mine. Based on the condition of high advancing speed, a method for determining the reasonable production capacity of open pit mines through time–space deduction simulation is proposed by Zhao et al.^24^. The theory of lag modelling approach was given by Ordin and Vasil'ev^25^ for optimizing the design capacity of an open pit gold mine to maximize the overall economic benefit over the mine's lifetime. The optimization findings on the open pit mine design capacity using the integral criteria of maximum economic indices utilizing the lag modelling approach are provided, and the influence of the market prices of gold on the optimized design capacity of the open pit mine is analyzed.

The above studies are open-pit metal mines as the research object, is to maximize the total net present value to dynamically adjust the annual production capacity. However, to ensure the annual coal supply for power generation and winter heating, the coal production of open-pit coal mines cannot be adjusted greatly every year. Therefore, the above research methods are not suitable for open-pit coal mines’ production capacity planning. At present, there is no effective simple method and formula for the planning of production capacity. Given this, this study analyses the relationship among the production capacity, the working face length and the annual advancing speed, and constructs the functional relationship model among the three for the first time. Taking Baorixile open-pit coal mine in Hulunbeier City, Inner Mongolia Autonomous Region, China as an example, four regions are divided according to the mining scope and coal seam development, and the capacity planning analysis is carried out by using the established relationship model. Then, proposed three plans for the mining district division. Finally, the optimal plan is obtained through index comparison, and on this basis, the reasonable production capacity of each mining district is determined.

## Model

**Figure 1:**
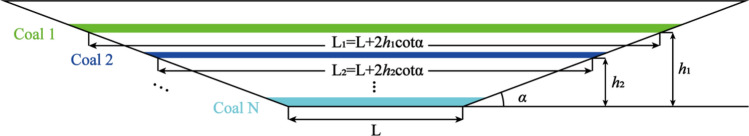
Spatial relationship of coal seams.

To plan the production capacity of open-pit coal mines, according to the occurrence of each coal seam in the mining boundary (as shown in Fig. 1), this paper studies the influence of working face length and advancing speed of working face on production capacity. The advancing speed of the working face depends on the operating efficiency of the mining equipment. At the same time, in the ‘Code for design of open pit mine of the coal industry, GB 50197-2015’ Article 2.4.5, it is stipulated that the advancing speed of the working face is not greater than 400 m/a when using a single bucket truck mining process^26^. Therefore, the advancing speed should be under these constraints, combined with the working face length to optimize production capacity.

According to the layer spacing and thickness of each coal seam, the distance $h_{i}$ between each coal seam and the bottom coal seam can be determined:

1 $$ h_{i} = \sum\limits_{i}^{n - 1} {d_{{i\left( {i + 1} \right)}} + \sum\limits_{i + 1}^{n} {m_{i} } } ,\;\;i = 1,2,...,n - 1 $$

When $i$ is the coal seam number; $h_{i}$ is the distance between coal $i$ and the bottom coal; $d_{{i\left( {i + 1} \right)}}$ is the distance between coal $i$ and coal $i{ + 1}$; $m_{i}$ is the average thickness of coal $i$.

According to the stability of the slope, the slope angle of the end slope is determined, and the interlayer distance of each coal seam in the mining range is determined according to the geological exploration results. Then, under the condition of determining the working face length of the bottom coal seam in the mining range, the working face length of other coal seams can be calculated. The calculation equation is as follows:

2 $$ L_{i} = L + 2h_{i} \cot \alpha $$

where $L_{i}$ is the working face length of coal $i$; $L$ is the working face length of the coal $n$; $\alpha$ is the angle of the pit.

Then, the capacity $Q$ can be expressed as:

3 $$ Q{ = }\sum\limits_{i = 1}^{n} {\left( {L_{i} \cdot m_{i} } \right)} \cdot V \cdot \rho $$

where $Q$ is the annual production capacity; $\rho$ is the volume weight of coal, $V$ is the annual advancing speed.

By substituting Eqs. (1) and (2) into Eq. (3):

4 $$ Q{ = }\left( {2\cot \alpha \sum\limits_{i = 1}^{n - 1} {m_{i} \left( {\sum\limits_{i}^{n - 1} {d_{{i\left( {i + 1} \right)}} + \sum\limits_{i + 1}^{n} {m_{i} } } } \right) + L \cdot \sum\limits_{i = 1}^{n} {m_{i} } } } \right) \cdot V \cdot \rho $$

It can be obtained from Eq. (4) that the production capacity is positively correlated with the working face length and the annual advancing speed. Under the condition that the production capacity is determined, the working face length is inversely proportional to the advancing speed^27^.

## Study area

**Figure 2:**
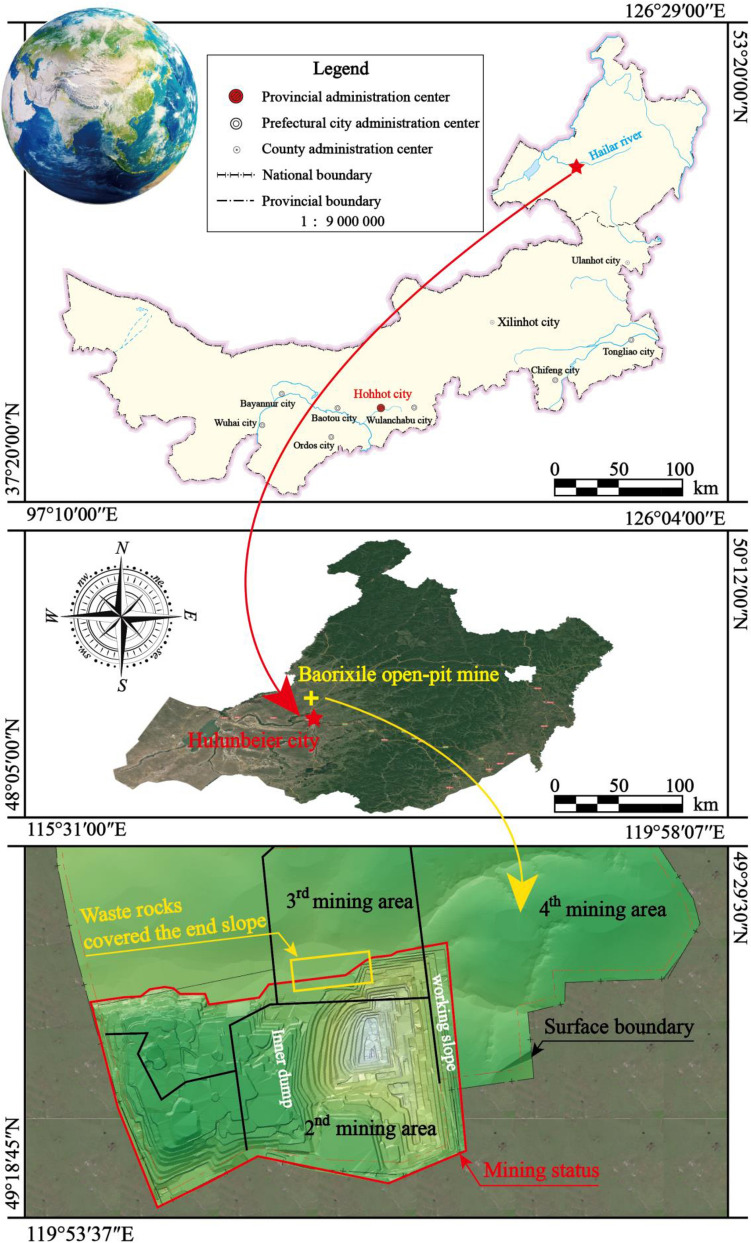
The geographic location of the study area.

The Chenbaerhuqi coal field in Hulunbuir City, Inner Mongolia Autonomous Region of China, is where the Baorixile open-pit coal mine is situated (as shown in Fig. 2)^7,28^. The mine's external perimeter spans a 50.72 km^2^ region, measuring 5.86 km in width from north to south and 10.98 km in length from east to west. The mine was constructed in 1998. The original design’s annual production capacity was 1.80 Mt. The reconstruction and expansion project passed the completion and acceptance of the National Energy Administration in December 2010, and 26.23 Mt coal resources were mined in 2011. In 2012, the mine successfully achieved a coal production capacity of 30.26 Mt/a through the stripping-mining-transportation-breaking-storage-loading integrated coal production system. Two sets of ground production systems achieve crushing coal 5000 t/h. In 2014, the approved production capacity of Baorixile Open-pit Coal Mine was 35 Mt/a.

Analysis of the mining status of the Baorixile open-pit coal mine in Fig. 2 shows that the main technical problems are: a large part of the north side of the 2nd mining district has been covered by the stripping waste rock of the inner dump, resulting in the turning plan, the 2nd mining district directly turns northward into the 3rd mining district is difficult to implement; The working slope of the 2nd mining district is advancing eastward to the mining boundary, then if continue to push eastward into the 4th mining district, the working slope is shortened by about 1500 m, it is difficult to achieve capacity targets. The original design of the mining district division plan and the corresponding mining procedure cannot be implemented, and cannot meet the requirements of 30–35 Mt/a capacity planning. Aiming at the capacity target of 30–35 Mt/a in Baorixile open-pit coal mine, this research studies the influence of working face length and annual advancing speed on production capacity redesigns the mining district division plan, and puts forward the advanced, economical, reasonable, safe and reliable mining district division and mining procedure plan of the Baorixile open-pit coal mine, to achieve the purpose of reducing the secondary stripping volume, shortening the transportation distance, reducing the stripping ratio and reliable connection of production capacity, to maximize the overall economic benefits of Baorixile open-pit coal mine during the mining period.

**Figure 3:**
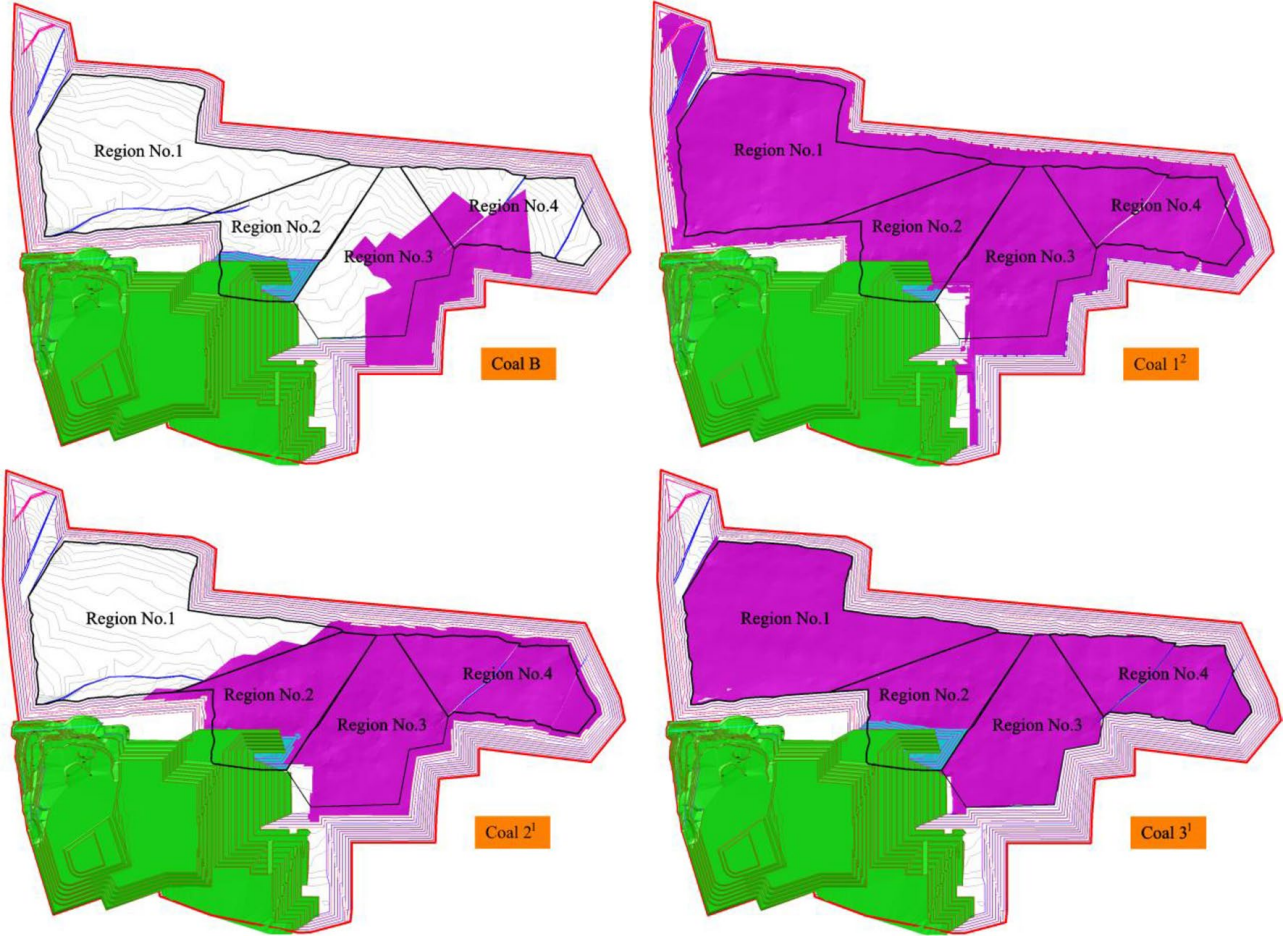
Distribution of each coal seam in the mining boundary.

There are coal B, coal 1^2^, coal 2^1^, and coal 3^1^ of Baorixile open-pit coal mine, and the distribution of each coal seam in the mining boundary is shown in Fig. 3. Coal B is only distributed in the southeast of the boundary, coal 1^2^ is developed in the whole area of the boundary, coal 2^1^ is distributed in the middle and east of the boundary, and coal 3^1^ is developed in the whole area of the boundary. According to the number and the thickness of coal seams, it is divided into four regions. Region No.1 has coal 1^2^ and coal 3^1^ that can be mined, Region No.2, Region No.3, and Region No.4 have coal 1^2^, coal 2^1^, and coal 3^1^ that can be mined. The study and analysis of the relationship among the working face length, production capacity and advancing speed of working face by dividing the region can provide an important basis for capacity planning.

Baorixile open-pit coal mine has typical characteristics of the composite coal seam, and the determination of the relationship among its working face length, advancing speed and production capacity relationship is more complex. Analysis of the relationship model established in the second chapter shows that according to the angle of the end slope and the interlayer spacing between coal seams, the working face length of each coal seam can be calculated, and their relationship can be determined. The angle of the end slope is 20° and the weight volume of coal is 1.16 t/m^3^ in Baorixile open-pit mine^29^.

### Relationship model among production capacity, working face length of coal 3^1^ and advancing speed in Region No.1

**Figure 4:**
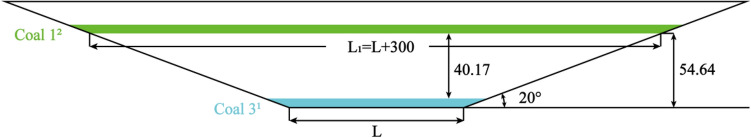
The spatial relationship of coal seams in Region No.1.

**Figure 5:**
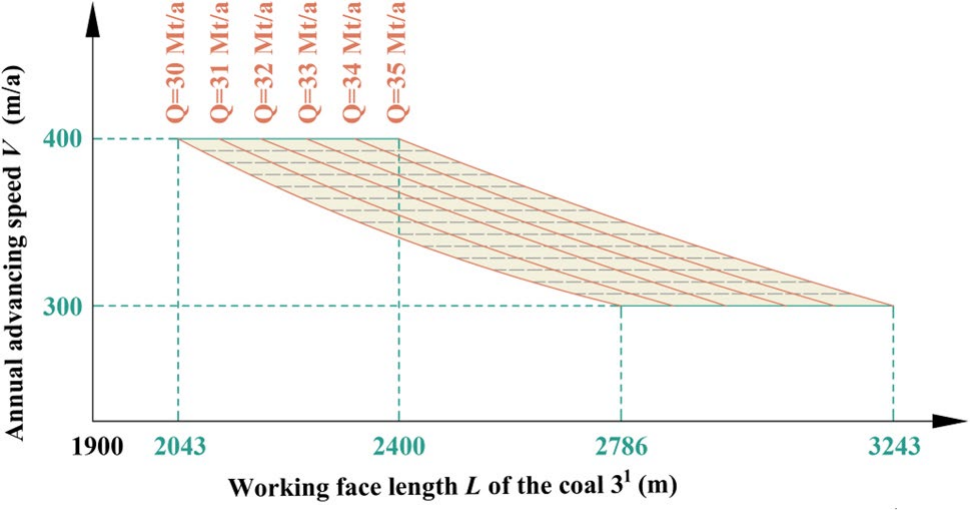
Relationship among production capacity, working face length of coal 3^1^ and advancing speed in Region No.1.

Region No. 1 offers the possibility of mining coal 1^2^ and coal 3^1^. Figure 4 illustrates the spatial relationship of coal seams, and Table 1 displays the thickness and spacing of coal seams. The relationship among production capacity, working face length of coal 3^1^, and advancing speed in Region No.1, as illustrated in Fig. 5 and Table 2 by Eq. (3).

**Table 1:** Coal seam information in Region No.1.

Coal seam	Average thickness (m)	Average spacing (m)
1^2^	18.69	40.17
3^1^	10.25

**Table 2:** Key parameters at capacities of 30 Mt/a and 35 Mt/a in Region No.1.

Annual advancing speed *V* (m/a)	Working face length *L* of the coal 3^1^ (m)	Production capacity *Q* (Mt/a)
300	2786	30
350	2348	30
400	2043	30
300	3243	35
350	2786	35
400	2400	35

By analyzing Fig. 5 and Table 2, it can be inferred that when both the length and advancing speed of coal 3^1^ working face fall within the shaded area depicted in Fig. 5, the production capacity will achieve *Q* = 30–35 Mt/a in Region No.1.

### Relationship model among production capacity, working face length of coal 3^1^ and advancing speed in Region No.2

**Figure 6:**
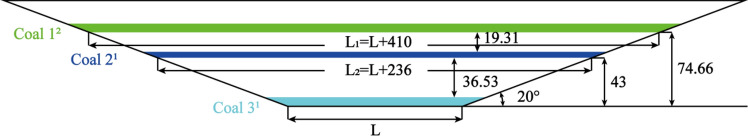
The spatial relationship of coal seams in Region No.2.

**Figure 7:**
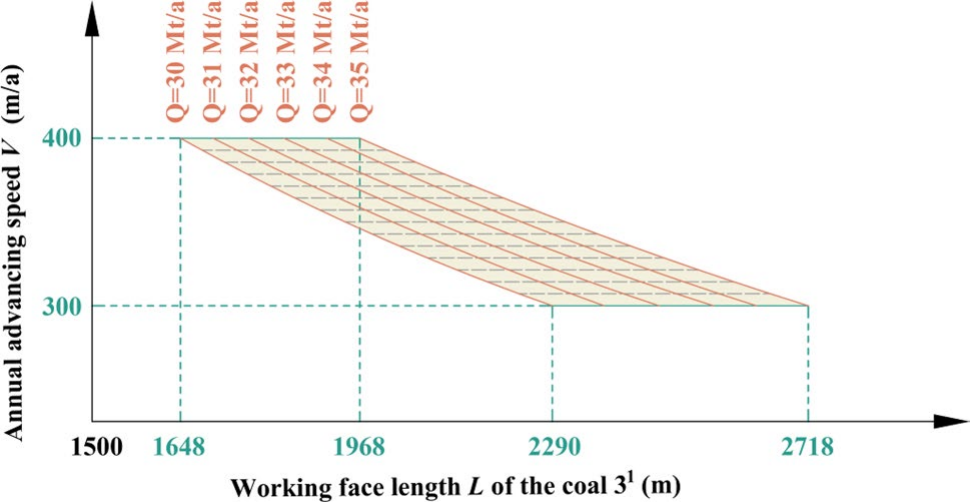
Relationship among production capacity, working face length and advancing speed of the coal 3^1^ in Region No.2.

Region No. 2 offers the possibility of mining coals 1^2^, coal 2^1^ and 3^1^. Figure 6 illustrates the spatial relationship of coal seams, and Table 3 displays the thickness and spacing of coal seams. The relationship among production capacity, working face length of coal 3^1^, and advancing speed in Region No.2, as illustrated in Fig. 7 and Table 4 by Eq. (3).

**Table 3:** Coal seam information in Region No.2.

Coal seam	Average thickness (m)	Average spacing (m)
1^2^	20.65	19.31
2^1^	4.06	
		36.53
3^1^	8.87

**Table 4:** Key parameters at capacities of 30 Mt/a and 35 Mt/a in Region No.2.

Annual advancing speed *V* (m/a)	Working face length *L* of the coal 3^1^ (m)	Production capacity *Q* (Mt/a)
300	2290	30
350	1924	30
400	1648	30
300	2718	35
350	2290	35
400	1968	35

By analyzing Fig. 7 and Table 4, it can be inferred that when both the length and advancing speed of coal 3^1^ working face fall within the shaded area depicted in Fig. 7, the production capacity will achieve *Q* = 30–35 Mt/a in Region No.2.

### Relationship model among production capacity, working face length of coal 3^1^ and advancing speed in Region No.4

**Figure 8:**
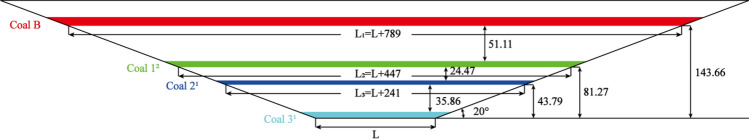
The spatial relationship of coal seams in Region No.3.

**Figure 9:**
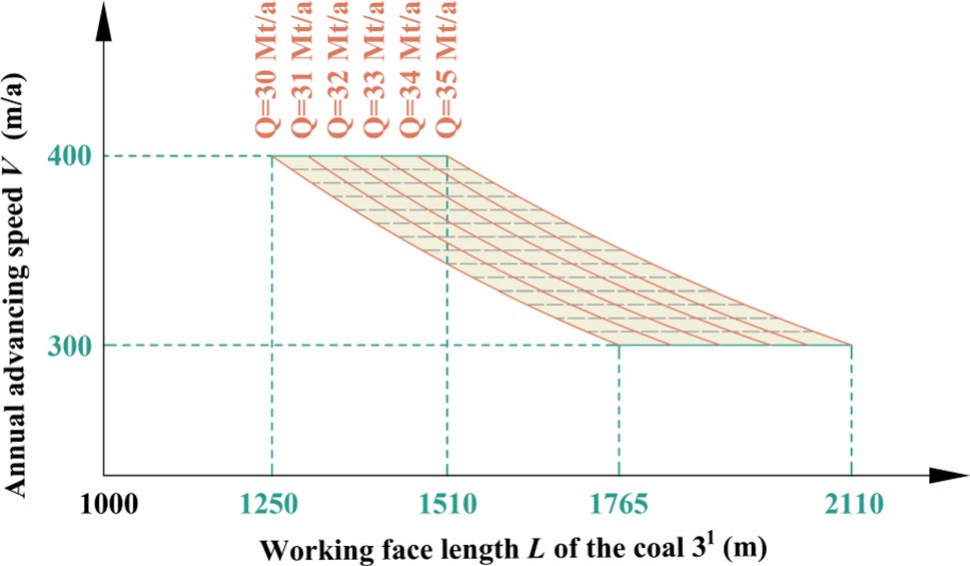
Relationship among production capacity, working face length and advancing speed of the coal 3^1^ in Region No.3.

Region No. 3 offers the possibility of mining coal B, coals 1^2^, coal 2^1^ and 3^1^. Figure 8 illustrates the spatial relationship of coal seams, and Table 5 displays the thickness and spacing of coal seams. The relationship among production capacity, working face length of coal 3^1^, and advancing speed in Region No.3, as illustrated in Fig. 9 and Table 6 by Eq. (3).

**Table 5:** Coal seam information in Region No.3.

Coal seam	Average thickness (m)	Average spacing (m)	Remark
B	1.81	51.11	Local occurrence of coal B in this region (average thickness 3.62 m * 1/2)
1^2^	24.18		
		24.47	
2^1^	7.09		
		35.86	
3^1^	8.76

**Table 6:** Key parameters at capacities of 30 Mt/a and 35 Mt/a in Region No.3.

Annual advancing speed *V* (m/a)	Working face length *L* of the coal 3^1^ (m)	Production capacity *Q* (Mt/a)
300	1765	30
350	1472	30
400	1250	30
300	2110	35
350	1765	35
400	1510	35

By analyzing Fig. 9 and Table 6, it can be inferred that when both the length and advancing speed of coal 3^1^ working face fall within the shaded area depicted in Fig. 9, the production capacity will achieve *Q* = 30–35 Mt/a in Region No.3.

### Relationship model among production capacity, working face length of coal 3^1^ and advancing speed in Region No.4

**Figure 10:**
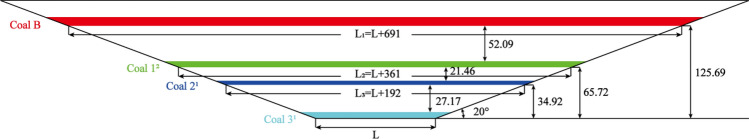
The spatial relationship of coal seams in Region No.4.

**Figure 11:**
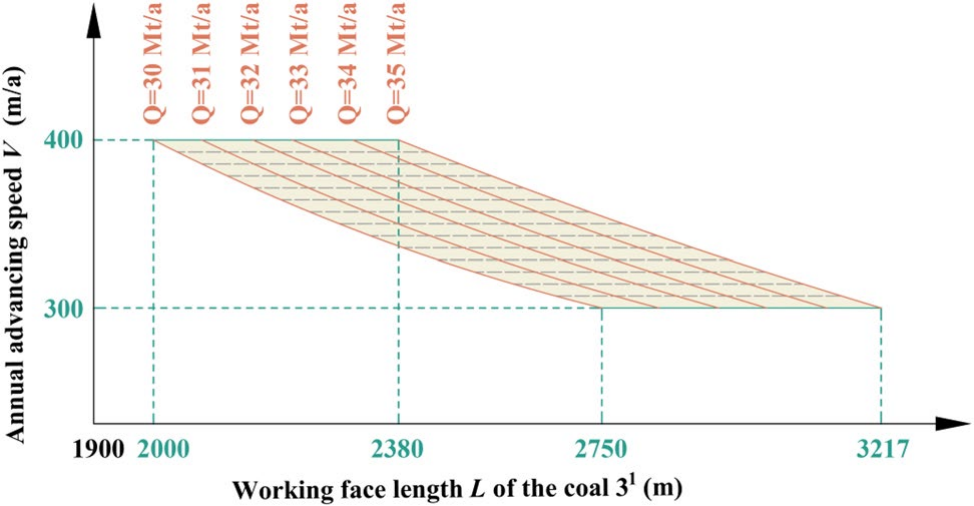
Relationship among production capacity, working face length and advancing speed of the coal 3^1^ in Region No.4.

Region No. 4 offers the possibility of mining coal B, coals 1^2^, coal 2^1^ and 3^1^. Figure 10 illustrates the spatial relationship of coal seams, and Table 7 displays the thickness and spacing of coal seams. The relationship among production capacity, working face length of coal 3^1^, and advancing speed in Region No.4, as illustrated in Fig. 11 and Table 8 by Eq. (3).

**Table 7:** Coal seam information in Region No.4.

Coal seam	Average thickness (m)	Average spacing (m)	Remark
B	1.57	52.09	Local occurrence of Coal B in this region (average thickness 3.93 m * 2/5)
1^2^	11.83		
		21.46	
2^1^	6.85		
		27.17	
3^1^	8.65

**Table 8:** Key parameters at capacities of 30 Mt/a and 35 Mt/a in Region No.4.

Annual advancing speed *V* (m/a)	Working face length *L* of the coal 3^1^ (m)	Production capacity *Q* (Mt/a)
300	2750	30
350	2329	30
400	2000	30
300	3217	35
350	2750	35
400	2380	35

By analyzing Fig. 11 and Table 8, it can be inferred that when both the length and advancing speed of coal 3^1^ working face fall within the shaded area depicted in Fig. 11, the production capacity will achieve *Q* = 30–35 Mt/a in Region No.4.

## Mining district division plans

**Figure 12:**
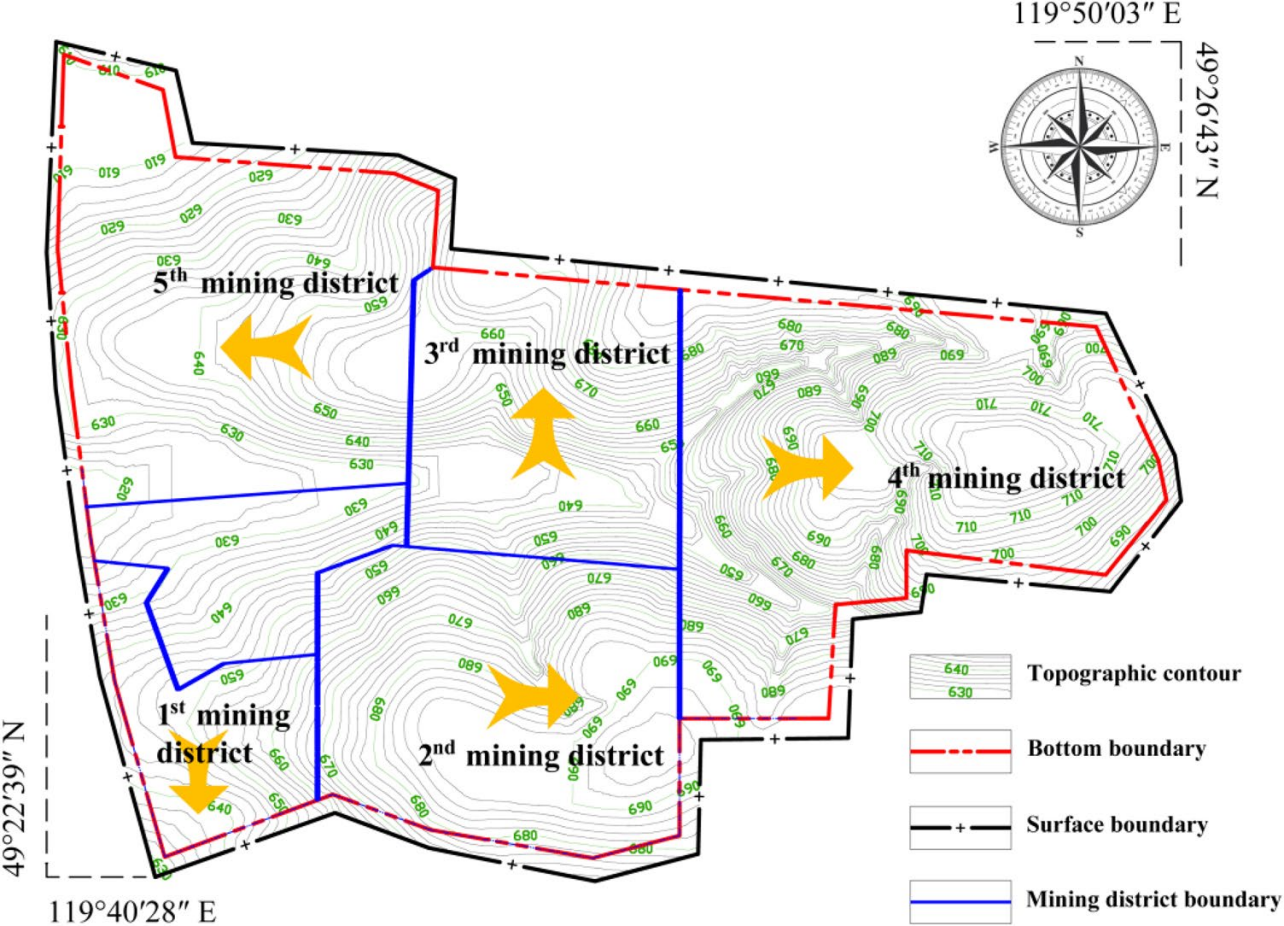
Mining district division plan of preliminary design in Baorixile open-pit coal mine.

To reduce the number of infrastructure projects and shorten the transport distance, large open-pit coal mines often adopt the method of mining by areas^30–33^. Width of each mining district (i.e. the working face length) to meet the requirements of production scale. The preliminary designed annual production capacity of the Baorixile open-pit mine is 10 Mt. On this basis, the mining district division plan is shown in Fig. 12. The mining procedure in the 1st mining district arranges a working face for east–west direction, advance from north to south; The mining procedure in the 2nd mining district arranges a working face for south-north direction, advance from east to west; The mining procedure in the 3rd mining district arranges working face for east–west direction, advance from south to north; The mining procedure in the 4th mining district arranges working face for south-north direction, advance from east to west; The mining procedure in the 5th mining district arranges working face for south-north direction, advance from west to east. The transition between mining districts is vertical steering. In the transition process of each mining district, from the 1st mining district to the 4th mining district, the method of leaving the ditch is adopted when the previous to the next, and the 5th mining district will be re-excavation ditch to mining.

In 2014, the approved production capacity of the Baorixile open-pit coal mines was 35 Mt/a. The coal mined is mainly sold to Heilongjiang Province and Jilin Province and bears the task of coal supply in Northeast China. Mine after years of development, the working face length of the main mining coal 1^2^ has reached 2600–3100 m, with an actual production capacity of about 30 Mt/a, but has not yet met the approved production capacity requirements (Table 9 illustrates the coal volume and stripping volume and working face development status in 2013–2019.). According to the data, it can be seen that the main mining coal seam is coal 1^2^, and the coal 2^1^ and 3^1^ working faces have not been fully developed. Therefore, it can be concluded that the open-pit coal mine has the potential to increase production capacity. The working face length can be changed by re-dividing the mining district, and the advancing speed can also be adjusted to achieve a production capacity of 35 Mt/a.

**Table 9:** Coal volume and stripping volume and working face development status in 2013–2019.

Year	Coal production (Mt)				Self-stripping (Mm^3^)	Outsourcing stripping (Mm^3^)	Total stripping volume (Mm^3^)	Working stripping ratio (m^3^/t)	Working face length of coal 1^2^ (m)	Advancing speed of coal 1^2^ (m)
	Coal 1^2^	Coal 2^1^	Coal 3^1^	Total						
2013	31.17	0.13	0.00	31.30	5.36	107.65	113.01	3.61	2798	–
2014	28.95	1.02	0.08	30.05	13.03	81.42	94.45	3.14	2810	193
2015	20.84	1.48	2.70	25.02	18.01	39.56	57.56	2.29	2738	229
2016	21.75	0.75	2.50	25.00	20.03	67.56	87.58	3.50	3144	181
2017	23.32	0.42	1.54	25.28	22.90	39.15	62.05	2.45	2655	230
2018	26.48	1.07	1.67	29.22	23.48	128.96	152.43	5.22	2806	301
2019	24.26	1.50	2.30	28.00	22.71	97.21	119.92	4.27	2709	371

The shape of the mining boundary in the remaining resource area of the Baorixile open-pit coal mine is extremely irregular, and the working face length changes greatly in the future mining process, which leads to the change of the advancing speed if the rated capacity is to be completed. Therefore, it is necessary to clarify the relationship among the working face length, the advancing speed and the production capacity, to divide the mining district reasonably. According to the thickness and distribution of each coal seam in the mining boundary, four regions are divided, and the relationship between the working face length, the advancing speed and the production capacity of each region are analyzed. The research results provide a basis for the study of the mining district division in the next part. On this basis, considering the factors such as production status, stripping ratio, fault influence, and the difficulty of transition between mining districts, the following three mining district division and mining procedure plans are proposed for the exploitation range of the remaining resource area of Baorixile open-pit coal mine.

### Mining district division plan 1

**Figure 13:**
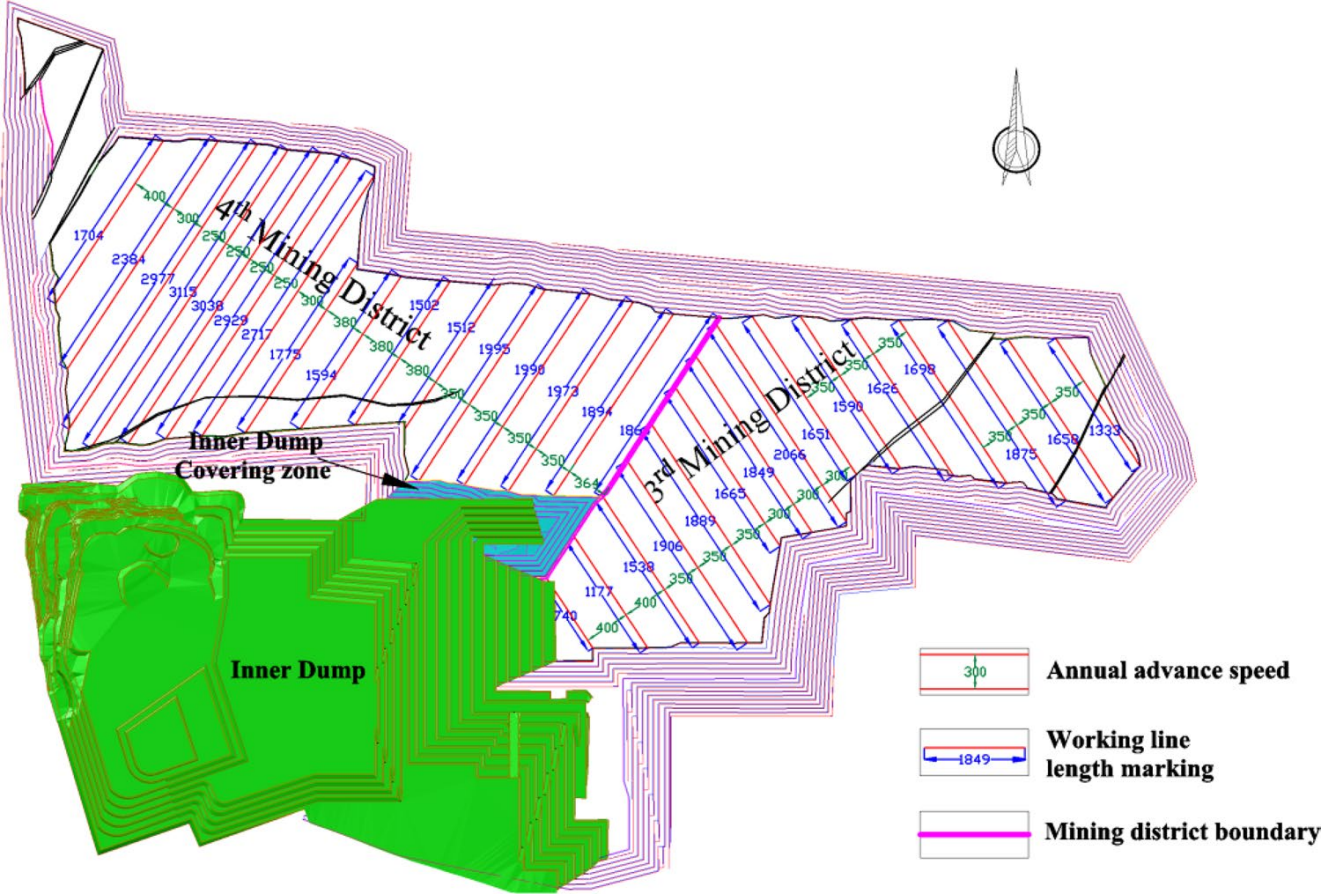
Mining district division Plan 1 and mining procedure.

In the mining area, the bottom line of the lowest bench on the east side of the north side of the 2nd mining district covered by the stripping waste rock is extended northward to the line formed by the north side as the boundary of the mining district, and the mining area is divided into two mining districts (as shown in Fig. 13). The eastern part of the mining district boundary line is the 3rd mining district, and the western part is the 4th mining district. To make the working face length meet the needs of the approved production capacity to be achieved, try to make the length of the different periods of the working face difference not so outrageous. The mining procedure in the 3rd mining district arranges an obliquely working face for northwest-southeast, parallel advance from southwest to northeast, and the mining procedure in the 4th mining district arranges an obliquely working face for southwest-northeast, parallel advance from southeast to northwest. The residual excavation volume in the mining boundary of mining district division Plan 1 is shown in Table 10.

**Table 10:** Residual excavation volume in mining boundary of mining district division plan 1.

Mining district		3rd mining district	4th mining district
Coal volume (Mt)	Coal B	26.76	0.00
	Coal 1^2^	229.65	279.18
	Coal 2^1^	82.01	14.18
	Coal 3^1^	76.64	131.22
Total coal volume (Mt)		415.06	424.59
Stripping volume (Mm^3^)		1743.62	1388.74
Stripping ratio (m^3^/t)		4.20	3.27

### Mining district division plan 2

**Figure 14:**
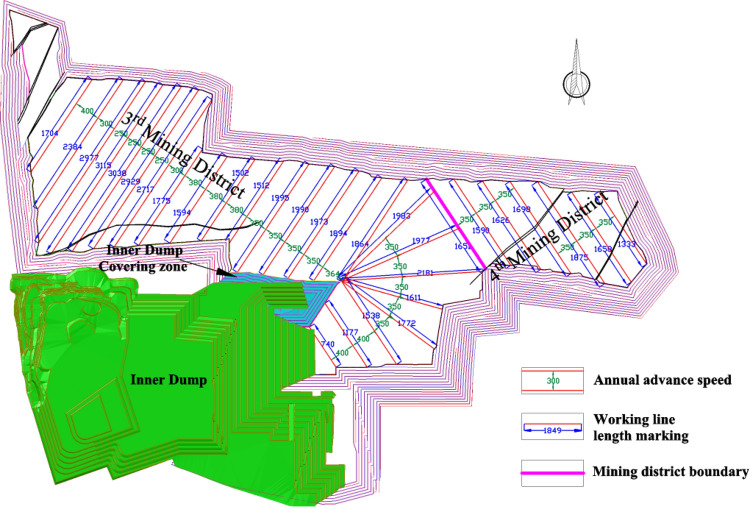
Mining district division Plan 2 and mining procedure.

In the mining area, the working face length of coal 3^1^ is more than 1500 m as the constraint condition, starting from the southeast convex slope of the final pit slope, a line is extended to the north side of the final pit to the northwest slope, and taking the extension line as the mining district boundary, the final pit is divided into two mining districts (as shown in Fig. 14). The east side of the line is the 4th mining district, and the west side is the 3rd mining district. To ensure that the working face length can meet the needs of the approved production capacity as far as possible, the working slope of the 3rd mining district is first advancing in parallel to the northeast, then turns to the northwest and finally develops parallel to the northwest. The working slope of the 4th mining district is advancing in parallel to the northeast. The residual excavation volume in the mining boundary of mining district division Plan 2 is shown in Table 11.

**Table 11:** Residual excavation volume in mining boundary of mining district division plan 2.

Mining district		3rd mining district	4th mining district
Coal volume (10 kt)	Coal B	2159	518
	Coal 1^2^	45,018	5865
	Coal 2^1^	6049	3570
	Coal 3^1^	17,690	3096
Total coal volume (10 kt)		70,916	13,049
Stripping volume (10 km^3^)		243,315	69,920
Stripping ratio (m^3^/t)		3.43	5.36

### Mining district division plan 3

**Figure 15:**
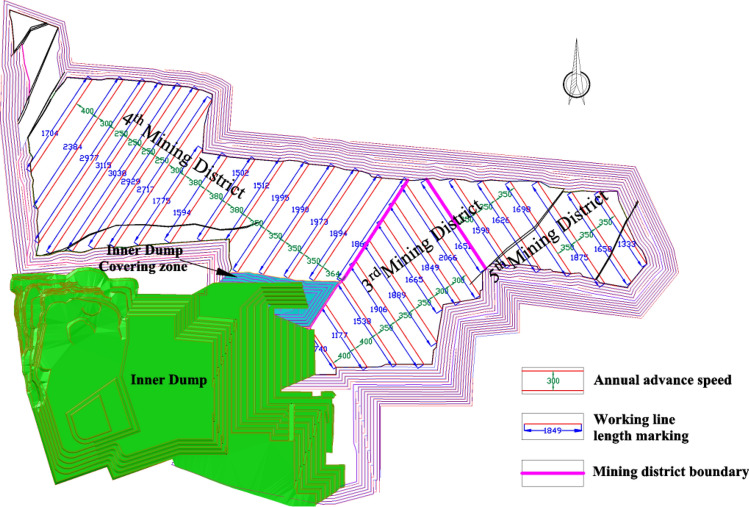
Mining district division Plan 3 and mining procedure.

In the mining area, the mining district division method of Plan 3 can be specifically described as taking the mining area boundary of Plan 1 as the boundary of 3rd mining district and 4th mining district and taking the mining district boundary of Plan 2 as the boundary of 3rd mining district and 5th mining district. And the final pit is divided into three mining districts (as shown in Fig. 15). To ensure that the working face length can meet the needs of the approved production capacity as far as possible, First, the working slope of the 3rd mining district advancing in parallel to the northeast, then the working slope of the 4th mining district advancing in parallel to the northwest, and final the working slope of the 5th mining district is advancing in parallel to the northwest. The residual excavation volume in the mining boundary of mining district division Plan 2 is shown in Table 12.

**Table 12:** Residual excavation volume in mining boundary of mining district division plan 3.

Mining district		3rd mining district	4th mining district	5th mining district
Coal volume (Mt)	Coal B	21.59	0.00	5.18
	Coal 1^2^	171.00	279.18	58.65
	Coal 2^1^	46.31	14.18	35.70
	Coal 3^1^	45.67	131.23	30.96
Total coal volume (Mt)		284.57	424.59	130.49
Stripping volume (Mm^3^)		1044.41	1388.74	699.21
Stripping ratio (m^3^/t)		3.67	3.27	5.36

### Comparison and analysis of plans

This study will analyze and compare the stripping ratio, the mining life, the influence of faults on mining, the difficulty of the transition of the mining districts, and the convenience of the transportation system layout to recommend a technically feasible and economical mining district division and mining procedure plan. Table 13 illustrates the specific situation of the comparative analysis of district division plans.

**Table 13:** Comparative analysis of district division plans.

Mining district division plans	Plan 1		Plan 2		Plan 3			Comparative result
	3rd mining district	4th mining district	3rd mining district	4th mining district	3rd mining district	4th mining district	5th mining district	
Coal volume (Mt)	415.06	424.59	709.16	130.49	284.57	424.59	130.49	Plan 3
Production life (a) (based on 30 Mt/a)	13.8	14.15	23.64	4.35	9.49	14.15	4.35	
Stripping ratio (m^3^/t)	4.20	3.27	3.43	5.36	3.67	3.27	5.36	
Effects of faults	Great	General	Small	Great	No	General	Great	Plan 3
Coal 2^1^ is overlaid by waste rock and coal pillar set for coal 3^1^		Exist		Exist			Exist	Similar
The convenience of the transportation system layout	General	Great	Great	General	Great	Great	General	Plan 3

All three plans are based on the same mining area, resulting in identical total recoverable coal volume and total mining life for each plan. However, the division of mining districts varies between plans, leading to differences in recoverable coal volume and mining life for each district.

After comparison, Plan 2’s third mining area has the largest recoverable coal volume at 709.16Mt/a and the smallest stripping ratio at 3.43m^3^/t. Its service life is also the longest at 23.64a, with relatively minor fault impact and a more convenient transportation system. However, its development direction is fan-shaped to the west, with uneven advancement speed along the working face direction. The maximum advancement speed exceeds 400 m/a^26^, which is technically challenging to achieve.

Plan 3 is similar to Plan 2, with the difference being that Plan 2’s third mining district is further divided into two districts. The working face development mode in Plan 2 is fan-shaped while that in Plan 3 is straight. Compared to the two plans, Plan 3’s working face advancement method is easier to achieve.

Plan 1’s third mining district has the largest stripping ratio and fault impact and its transportation system layout is difficult to connect, making it unsuitable for division planning.

After a comprehensive comparison, Plan 3 is adopted as the division plan for the remaining mining resources in the Baorixile open-pit coal mine.

## Production capacity planning

Coal seam number, coal seam thickness, mining district width, and advancing speed are key factors to control production capacity. However, these elements vary greatly in various mining districts. It is necessary to analyze and determine the production capacity range of each mining district of the recommended plan 3 into different Regions according to the relationship model of the working face length, production capacity and advancing speed of each district determined by the above research.

### Production capacity of the 3rd mining district

**Figure 16:**
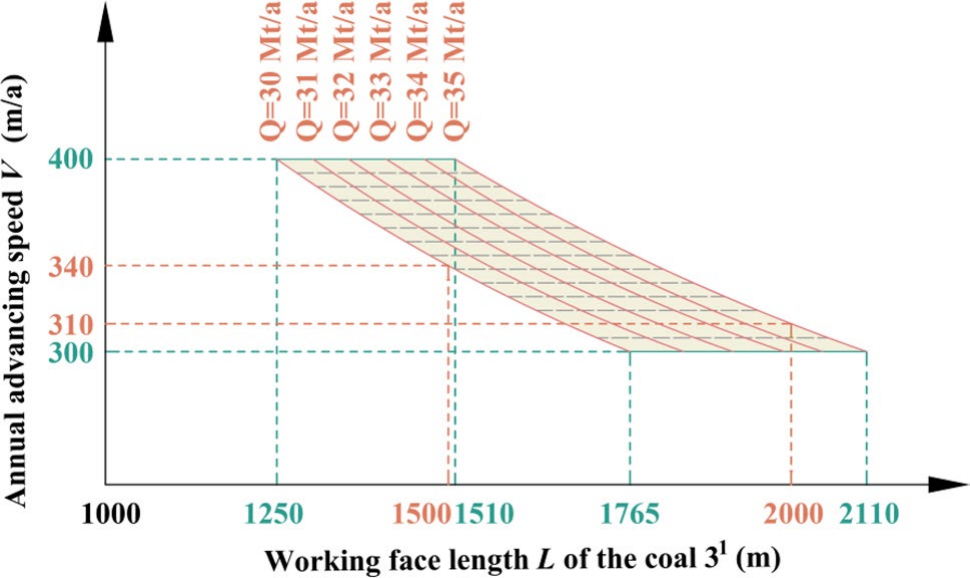
The productivity relationship in the 3rd mining district.

The 3rd mining district is within the scope of Region No.3, the coal seams that can be mined are coal B, coal 1^2^, coal 2^1^ and coal 3^1^, and the working face length of coal 3^1^ is between 1500 and 2000 m. Through the relationship model of Region No.3, the relationship of the working face length of the coal 3^1^, advancing speed, and production capacity in the 3rd mining district is shown in Fig. 16. When the advancing speed is between 310 and 400 m/a, a production capacity of 30 Mt/a to 35 Mt/a can be achieved. The coal seams in this area are the thickest, the working face length does not change much, and the productivity is relatively stable.

### Production capacity of the 4th mining district

**Figure 17:**
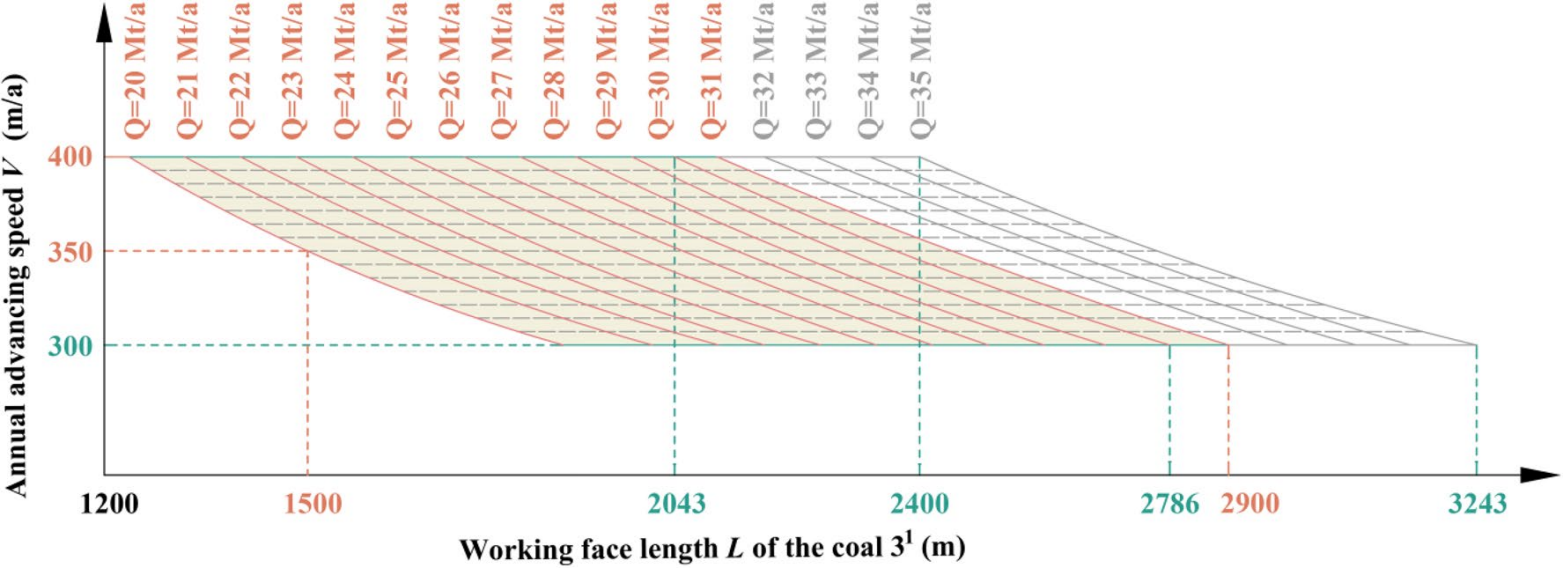
The productivity relationship in the 4th mining district distributed in Region No.1.

The 4th mining district is distributed in Region No.1 and No.2, while in Region No.1, the coal seams that can be mined are coal 1^2^ and coal 3^1^, and the working face length of coal 3^1^ is between 1500 and 2900 m. Through the relationship model of Region No.1, the productivity relationship in the 4th mining district distributed in Region No.1 is shown in Fig. 17. When the advancing speed is between 350 and 400 m/a, a production capacity of 20 Mt/a to 31 Mt/a can be achieved. The coal seam in this area is obviously thinner, the working face length changes greatly, and the productivity fluctuates greatly. It is also difficult to stabilize productivity by adjusting the recommended strength.

**Figure 18:**
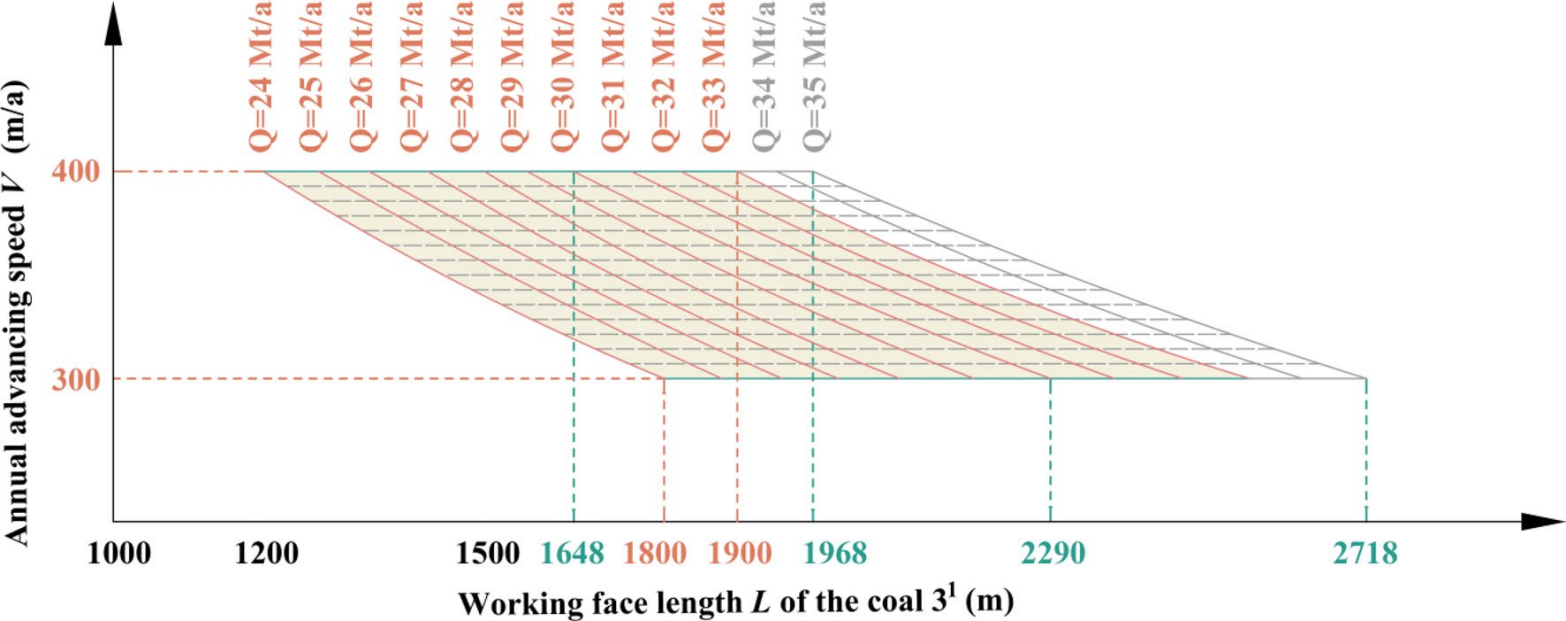
The productivity relationship in the 4th mining district distributed in Region No.2.

When in Region No.2, the coal seams that can be mined are coal 1^2^ coal 2^1^, and coal 3^1^, and the working face length of coal 3^1^ is between 1800 and 1900 m. Through the relationship model of Region No.2, the productivity relationship in the 4th mining district distributed in Region No.2 is shown in Fig. 18. When the advancing speed is between 300 and 400 m/a, a production capacity of 24 Mt/a to 33 Mt/a can be achieved. The coal seam in this area is thick, the working face length changes little, and the productivity can be stabilized by adjusting the recommended strength.

### Production capacity of the 5th mining district

**Figure 19:**
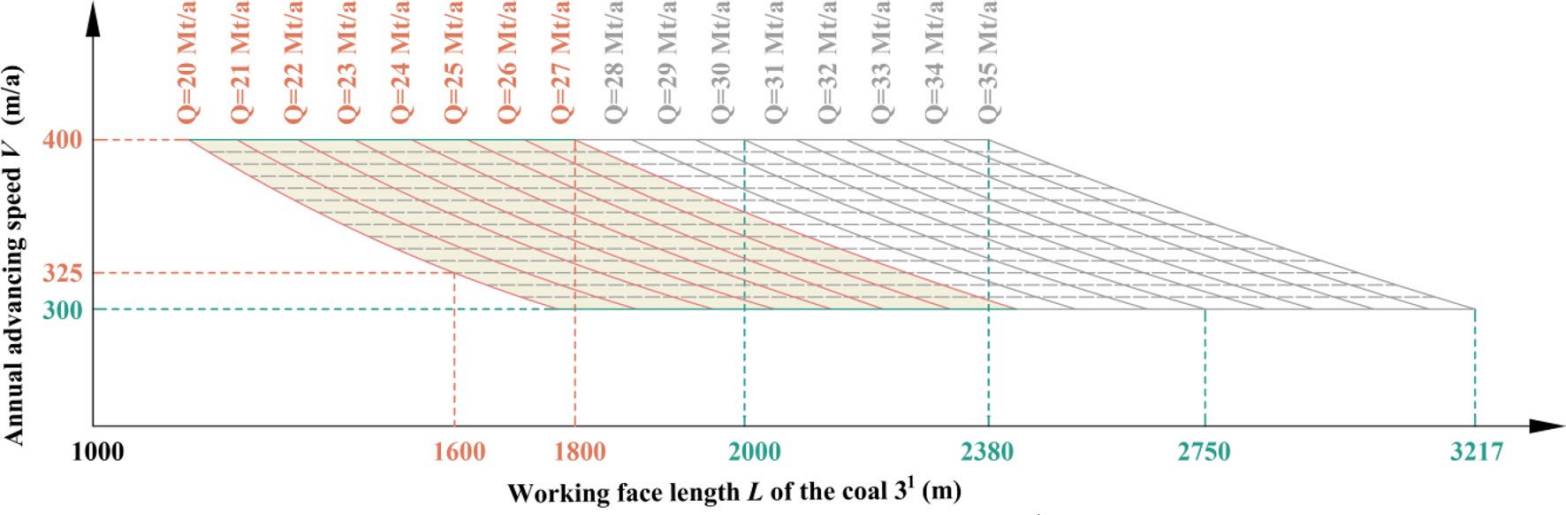
The productivity relationship in the 5th mining district.

The 5th mining district is within the scope of Region No.4, the coal seams that can be mined are coal B, coal 1^2^, coal 2^1^ and coal 3^1^, and the working face length of coal 3^1^ is between 1600 and 1800 m. Through the relationship model of Region No.4, the productivity relationship in the 5th mining district is shown in Fig. 19. When the advancing speed is between 325 and 400 m/a, a production capacity of 20 Mt/a to 37 Mt/a can be achieved. The coal seam in this area is also obviously thinner, the working face length is small as a whole, and the productivity can be stabilized by adjusting the propulsion strength.

The parameter indexes and capacity planning results for each mining district of the recommended Plan 3 are shown in Table 14.

**Table 14:** The parameter indexes and capacity planning results for each mining district of the recommended Plan 3.

Mining district		3rd mining district				4th mining district					5th mining district			
Region		Region No.3				Region No.1		Region No.2			Region No.4			
Coal seam		B	1^2^	2^1^	3^1^	1^2^	3^1^	1^2^	2^1^	3^1^	B	1^2^	2^1^	3^1^
Average thickness (m)	Single	1.81	24.18	7.09	8.76	18.69	10.25	20.65	4.06	8.87	1.57	11.83	6.85	8.65
	Total	41.84				28.94		33.58			28.90			
Working face length (m)		1500–2000				1500–2900		1800–1900			1600–1800			
Annual advancing speed (m/a)		310–400				350–400		300–400			325–400			
Production capacity (Mt/a)		30–35				20–31		24–33			20–27			

## Discussion

The production capacity planning method proposed in this study takes into account the influence of the irregular shape of mining boundary on the working face length. It can carry out long-term capacity planning for the full life cycle of the mine by preparing scheduling tasks of production equipment and project deployment in advance based on the production capacity range of each divided area. The proposed method conforms to the actual production situation of open-pit mine and has broad application prospects.

However, due to the irregularity of the mining boundary, there will be large fluctuations in the production capacity of the same region, even though there is little fluctuation in the advancing speed of the working face. The function model established in this paper can only give the range of production capacity in the divided region, but it cannot obtain more refined production capacity for short-term production planning.

Therefore, we will carry out a further study based on the results of this study in the future: consider the efficiency of operating equipment and the slope angle, we will carry out more detailed dynamic planning for the production capacity in each region.

## Conclusion

1. This study establishes a relationship model between the working face length, the annual advancing speed, and production capacity based on the spatial relationship between the coal seams in the final pit of the open-pit coal mine.
2. Taking the Baorixile Open-pit Coal Mine in the northeast of China’s Inner Mongolia Autonomous Region as the research area, the remaining unmined parts are divided into four regions based on the distribution, development and shape of the coal seams in the final pit of the mine. Using the relationship model established in this paper and under the condition of ensuring production capacity and stable production continuity, the relationship model between the working face length, the annual advancing speed and the production capacity of each region is constructed.
3. Based on the relationship model in each region, and considering the factors such as production status, stripping ratio, fault influence, and the difficulty of transition between mining districts, the three mining district division and mining procedure plans are proposed for the exploitation range of the remaining resource area of the Baorixile open-pit coal mine.
4. Carry out production capacity planning for Plan 3, and determine that the production capacity of the 3rd mining district is determined to be 30–35 Mt/a; the production capacity of the 4th mining district in Region 1 is 20–31 Mt/a, and the production capacity in Region 2 is 24–33 Mt/a; the production capacity of the 5th mining district is 20–27 Mt/a.

## Data Availability

The data used and analysed during the current study available from the corresponding author on reasonable request.
